# Healthcare-Associated Legionnaires’ Disease, Europe, 2008−2017

**DOI:** 10.3201/eid2610.181889

**Published:** 2020-10

**Authors:** Julien Beauté, Diamantis Plachouras, Sven Sandin, Johan Giesecke, Pär Sparén

**Affiliations:** European Centre for Disease Prevention and Control, Stockholm, Sweden (J. Beauté, D. Plachouras);; Karolinska Institutet, Stockholm (J. Beauté, S. Sandin, J. Giesecke, P. Sparén);; Icahn School of Medicine at Mount Sinai, New York, New York, USA (S. Sandin);; Seaver Autism Center for Research and Treatment at Mount Sinai, New York (S. Sandin)

**Keywords:** Legionnaires’ disease, healthcare-associated, Europe, surveillance, Legionella, bacteria, respiratory infections, Legionella pneumophila

## Abstract

Healthcare-associated Legionnaires’ disease (HCA LD) can cause nosocomial outbreaks with high death rates. We compared community-acquired LD cases with HCA LD cases in Europe during 2008−2017 using data from The European Surveillance System. A total of 29 countries reported 40,411 community-acquired and 4,315 HCA LD cases. Of the HCA LD cases, 2,937 (68.1%) were hospital-acquired and 1,378 (31.9%) were linked to other healthcare facilities. The odds of having HCA LD were higher for women, children and persons <20 years of age, and persons >60 years of age. Out of the cases caused by *Legionella pneumophila* with a known serotype, community-acquired LD was more likely to be caused by *L. pneumophila* serogroup 1 (92.3%) than was HCA LD (85.1%). HCA LD patients were more likely to die. HCA LD is associated with specific patient demographics, causative strains, and outcomes. Healthcare facilities should consider these characteristics when designing HCA LD prevention strategies.

Legionnaires’ disease (LD) is a severe pneumonia caused by *Legionella*, a genus of gram-negative bacteria found in aquatic environments and human-made water systems (*1*). LD is a notifiable condition in all 30 European Union (EU) and European Economic Area (EEA) countries, where »70% of reported cases are community-acquired, »20% are travel-associated, and »10% are healthcare-associated (HCA) (*2*). In 2015, HCA LD accounted for 20% of all cases in the United States reported to the Centers for Disease Control and Prevention (*3*). The overall EU–EEA LD notification rate increased during 2011–2017 for unknown reasons (*2*,*4*).

Public health professionals should not overlook HCA LD; although it is relatively uncommon, it is associated with nosocomial outbreaks, underdiagnosis, and a high death rate of »30% (*5*–*7*). During 2006–2017, nearly 25% of identified outbreaks in the United States and several countries in Europe occurred in hospital or healthcare settings (*6*). During 2005–2009 in the United Kingdom and 2008–2010 in Spain, »3%–4% of HCA pneumonia cases were caused by *Legionella* (*8*,*9*). Hospital patients and residents of long-term care facilities are more likely to have LD risk factors, such as older age, chronic conditions, history of organ transplantation, or immunodeficiency (*7*). As such, hospital patients and residents of long-term care facilities might be more susceptible to *Legionella* (*10*).

Inhalation and aspiration are major modes of HCA LD transmission (*11*); potable water is a common source of infection (*7*). Because *Legionella* can colonize hospital water systems, possible sources of nosocomial infection include bathing, steam-heated towels, humidifiers, decorative fountains, and some medical devices (*12*,*13*). In children, HCA LD has been reported in association with heated birthing pools (*14*). HCA LD can be prevented by reducing the colonization of *Legionella* in hospitals (*15*). We describe the epidemiology of HCA LD in Europe using EU surveillance data to determine its differences from community‐acquired LD in terms of seasonality, demographics, causative pathogens, and outcomes.

## Methods

### LD Data

The European Legionnaires’ disease Surveillance Network, which comprises Iceland, Norway, and all 28 EU member states, including the United Kingdom, operates under the European Centre for Disease Prevention and Control (Stockholm, Sweden). Each state annually reports its LD cases to The European Surveillance System database hosted by the European Centre for Disease Prevention and Control. Countries report their LD cases with variables such as patient age, patient sex, date of disease onset, probable setting of infection (e.g. travel-associated), whether the case-patient is part of a cluster, laboratory method used for diagnosis, causative pathogen, and clinical outcome. LD patients who travelled (abroad or domestically) 2–10 days before disease onset are considered to have travel-associated LD. Many EU–EEA countries define HCA LD on the basis of whether the patient was in a hospital or healthcare facility <10 days before disease onset (*16*–*18*). Community-acquired LD is a diagnosis of exclusion (i.e., non-HCA, non–travel-associated). We defined a locally acquired case as any case not associated with travel.

Our analysis included all locally acquired cases reported during 2008–2017 that met the 2012 EU–EEA case definition of confirmed and probable cases of LD (*19*). We excluded travel-associated cases because they encompass heterogeneous exposures. We reclassified LD cases reported before 2012 according to the 2012 EU–EEA case definition. We defined hospital-acquired cases as those reported from a hospital, whereas HCA LD cases comprised hospital-acquired cases and cases reported from other healthcare facilities (e.g., nursing homes). We made this distinction for 2 main reasons. First, hospital patients, independent of age, might be immunocompromised and therefore more susceptible to LD. Second, the duration of *Legionella* exposure might be shorter for patients admitted to the hospital for acute care than for residents of long-term care facilities.

### Statistical Analyses

We compared the characteristics of HCA LD patients and community-acquired LD patients. We sorted patients into 8 groups by age at diagnosis (*2*). We compared their characteristics by using the χ^2^ test with a 2-sided p value of <0.05. In addition, we used logistic regression to analyze the odds of acquiring HCA or community-acquired LD, the odds of death, and the confounding effects of age and sex.

In a subanalysis of culture-confirmed cases (i.e., cases ascertained by isolation of *Legionella* spp. from respiratory secretions or any normally sterile site), we compared the causative pathogens of HCA LD patients with community-acquired LD patients. We grouped *Legionella* isolates by species and monoclonal subtypes; we further classified monoclonal subtypes by the virulence-associated epitope recognized by monoclonal antibody (mAb) 3/1 of the Dresden Panel (*10*). We further explored the factors associated with outcome in a subset analysis of culture-confirmed cases with information about the causative strain. We used Stata 14 (StataCorp LLC, https://www.stata.com) for all statistical analyses.

## Results

During 2008−2017, a total of 30 countries in Europe reported 64,409 LD cases. We excluded 446 (0.7%) of these case-patients because a laboratory method for diagnosis was not reported. We further excluded 6,788 (10.5%) LD patients, including all case-patients from Sweden, because setting of infection was not reported. These exclusions resulted in a preliminary analysis dataset of 57,175 LD patients. Of these, LD for 40,411 (70.7%) were reported as community-acquired, 11,512 (20.1%) as travel-associated, 4,315 (7.5%) as HCA, and 937 (1.6%) as associated with other settings. We then excluded travel-associated cases and those associated with other settings, resulting in our analysis dataset of 44,726 LD patients reported by 29 countries, of whom 40,411 (90.4%) had community-acquired LD and 4,315 (9.6%) had HCA LD (Table 1).

**Table 1:** Locally acquired cases of Legionnaires' disease, European Union–European Economic Area, 2008−2017

Country	Community-acquired cases, no. (%)	Healthcare-associated cases, no. (%)	Total, no. (%)
Austria	841 (2.1)	63 (1.5)	904 (2.0)
Belgium	234 (0.6)	72 (1.7)	306 (0.7)
Bulgaria	9 (<0.1)	1 (<0.1)	10 (<0.1)
Croatia*	124 (0.3)	6 (0.1)	130 (0.3)
Cyprus	0	9 (0.2)	9 (<0.1)
Czech Republic	505 (1.2)	44 (1.0)	549 (1.2)
Denmark	813 (2.0)	137 (3.2)	950 (2.1)
Estonia	33 (0.1)	20 (0.5)	53 (0.1)
Finland	27 (0.1)	4 (0.1)	31 (0.1)
France	8,564 (21.2)	1,571 (36.4)	10,135 (22.7)
Germany	3,197 (7.9)	290 (6.7)	3,487 (7.8)
Greece	182 (0.5)	28 (0.6)	210 (0.5)
Hungary	48 (0.1)	101 (2.3)	149 (0.3)
Iceland	9 (<0.1)	4 (0.1)	13 (<0.1)
Ireland	44 (0.1)	5 (0.1)	49 (0.1)
Italy	11,307 (28.0)	1,192 (27.6)	12,499 (27.9)
Latvia	231 (0.6)	0	231 (0.5)
Lithuania	18 (<0.1)	6 (0.1)	24 (0.1)
Luxembourg	1 (<0.1)	2 (<0.1)	3 (<0.1)
Malta	18 (<0.1)	1 (<0.1)	19 (<0.1)
Netherlands	2,129 (5.3)	58 (1.3)	2,187 (4.9)
Norway	117 (0.3)	0	117 (0.3)
Poland	24 (0.1)	19 (0.4)	43 (0.1)
Portugal	1,096 (2.7)	62 (1.4)	1,158 (2.6)
Romania	8 (<0.1)	1 (<0.1)	9 (<0.1)
Slovakia	52 (0.1)	9 (0.2)	61 (0.1)
Slovenia	653 (1.6)	1 (<0.1)	654 (1.5)
Spain	8,352 (20.7)	501 (11.6)	8,853 (19.8)
United Kingdom	1,775 (4.4)	108 (2.5)	1,883 (4.2)
Total	40,411 (100.0)	4,315 (100.0)	44,726 (100.0)

*Croatia started reporting Legionnaires’ disease in 2013.

**Figure 1:**
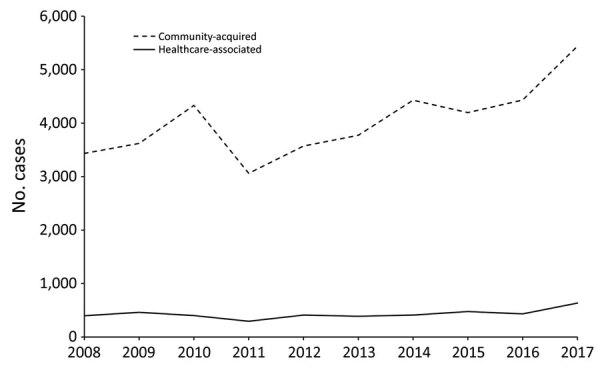
Locally acquired cases of Legionnaires’ disease, European Union–European Economic Area, 2008–2017. Not included are data from Croatia, which started reporting Legionnaires’ disease in 2013.

The annual number of locally acquired LD cases fluctuated from 3,357 cases in 2011 to 6,074 in 2017 (Figure 1). During 2011−2017, diagnoses of community-acquired LD and HCA LD increased. The average proportion of HCA LD among all LD cases was 10.7%, fluctuating between 9.3% in 2010 and 12.7% in 2009.

The highest proportions of HCA LD cases occurred in countries that reported <200 locally acquired cases. In Cyprus, Estonia, Hungary, Iceland, Luxembourg, and Poland, this proportion was >25%. Of the countries that reported >200 cases, the highest proportions of HCA LD occurred in Belgium (23.5%), France (15.5%), and Denmark (14.4%). Latvia and Norway did not report any HCA LD cases. Of the 4,315 HCA LD cases, 2,937 (68.1%) were hospital-acquired and 1,378 (31.9%) were linked to other healthcare facilities. Confirmation was slightly higher for community-acquired cases than for HCA LD (94.8% vs. 94.1%; p<0.05). France and Italy reported 2,763 (64.0%) HCA LD cases.

### Demographic Data

Of the 4,310 HCA LD patients for whom sex was known, 2,499 (58.0%) were male, resulting in a male:female ratio of 1.4:1. However, the proportion of HCA LD was higher for female LD patients than for male LD patients (14.2% vs. 7.8%; p<0.01). The male:female ratio was lower (0.9:1) for both younger (<20 years of age) and older (>80 years of age) patients; the ratio peaked at 2.2:1 for patients 40–49 years of age. Of the 4,313 HCA LD patients for whom age was known, 2,650 (61.4%) were >70 years of age. The proportion of HCA LD patients >70 years of age was higher for patients linked to other healthcare facilities than for those linked to hospitals (80.0% vs. 52.8%; p<0.01). After adjustment for age, year, and reporting country, women were more likely than men to have acquired their infection in a healthcare facility (odds ratio [OR] 1.60, 95% CI 1.49–1.71) (Table 2). Patients <20 years of age of were twice as likely as patients 50–59 years of age to have HCA LD (OR 2.04, 95% CI 1.25–3.33). The risk for an HCA LD diagnosis increased with age for patients >60 years of age, peaking for patients >80 years of age (OR 4.58, 95% CI 4.11–5.12).

**Table 2:** Main characteristics of locally acquired cases of Legionnaires' disease with adjusted predictors of healthcare-associated Legionnaires' disease, European Union–European Economic Area, 2008−2017*

Characteristic	Community-acquired cases, no. (%)	Healthcare-associated cases, no. (%)	Univariate logistic regression, OR (95% CI)	Multivariable logistic regression, OR (95% CI)†
Total	40,411 (100.0)	4,315 (100.0)		
Sex				
M	29,411 (73.0)	2,499 (58.0)	Referent	Referent
F	10,899 (27.0)	1,811 (42.0)	1.96 (1.83–2.09)	1.60 (1.49–1.71)
Unknown	101	5	Not included	Not included
Age at diagnosis, y				
<20	167 (0.4)	32 (0.7)	3.55 (2.41–5.24)	2.04 (1.25–3.33)
20–29	645 (1.6)	33 (0.8)	0.95 (0.66–1.36)	0.84 (0.57–1.23)
30–39	2,099 (5.2)	85 (2.0)	0.75 (0.59–0.95)	0.68 (0.53–0.87)
40–49	5,603 (13.9)	244 (5.7)	0.81 (0.69–0.94)	0.83 (0.71–0.98)
50–59	9,233 (22.9)	498 (11.5)	Referent	Referent
60–69	8,858 (21.9)	771 (17.9)	1.61 (1.44–1.81)	1.65 (1.46–1.86)
70–79	7,626 (18.9)	1,042 (24.2)	2.53 (2.27–2.83)	2.57 (2.29–2.88)
>80	6,127 (15.2)	1,608 (37.3)	4.87 (4.38–5.41)	4.58 (4.11–5.12)
Unknown	53	2	Not included	Not included
Cluster status			Not tested	Not tested
Sporadic	27,609 (94.0)	2,325 (89.4)	Not tested	Not tested
Clustered	1,764 (6.0)	275 (10.6)	Not tested	Not tested
Unknown	11,038	1,715	Not tested	Not tested
Culture confirmation			Not tested	Not tested
Yes	4,200 (10.4)	684 (15.9)	Not tested	Not tested
No	36,211 (89.6)	3,631 (84.1)	Not tested	Not tested
Outcome			Not tested	Not tested
Alive	26,630 (91.4)	2,301 (71.2)	Not tested	Not tested
Dead	2,518 (8.6)	930 (28.8)	Not tested	Not tested
Unknown	11,263	1,084	Not tested	Not tested

*OR, odds ratio. †Adjusted for year and reporting country.

### Seasonality

**Figure 2:**
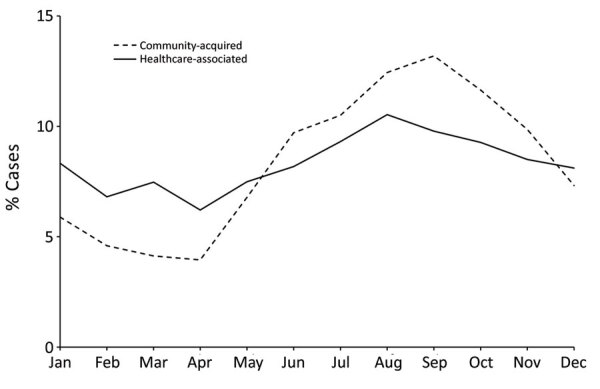
Timing of onset of locally acquired Legionnaires’ disease cases, European Union–European Economic Area, 2008–2017. Not included are data from Croatia, which started reporting Legionnaires’ disease in 2013.

The monthly distribution of onset peaked in August and September for both community-acquired and HCA LD (Figure 2). The proportion of community-acquired LD cases that developed during June–November was greater than that of HCA LD (66.9% vs. 55.8%; p<0.01).

### Laboratory Test Results

During 2008–2017, The European Surveillance System recorded 48,197 laboratory test results for the 44,726 LD patients included in this analysis. LD diagnosis by urinary antigen test (UAT) was more common for community-acquired than HCA LD cases (88.7% vs. 84.3%; p<0.01). On the other hand, culture confirmation of LD was more common for HCA LD than community-acquired cases (15.9% vs. 10.4%; p<0.01) (Table 2). Of the 4,884 culture-confirmed cases, 2,205 (45.1%) were also ascertained by UAT.

Among HCA LD cases, the proportion of culture-confirmed cases was higher for hospital-acquired cases than cases linked to other healthcare settings (18.8% vs. 9.7%; p<0.01). PCR diagnosis was more likely for HCA LD than for community-acquired LD (6.8% vs. 5.0%; p<0.01). For both community-acquired and HCA LD, the proportion of cases ascertained on the basis of a single high titer of a specific serum antibody was similar (»2.5%). The proportion of cases diagnosed by a 4-fold rise in titer or by direct immunofluorescence assay also was similar for both groups (<1% for both tests).

### Pathogens

Of the 4,859 culture-confirmed cases reported with a known causative pathogen species, 4,739 (97.5%) were caused by *Legionella pneumophila*. This proportion was similar for community-acquired and HCA LD cases (97.4% vs. 98.1%; p = 0.31) (Table 3). Of the 4,533 laboratory-confirmed cases of *L. pneumophila* reported with a known serogroup, 4,137 (91.3%) were caused by *L. pneumophila* serogroup 1. This proportion was higher for community-acquired cases than HCA LD cases (92.3% vs. 85.1%; p<0.01). Following *L. pneumophila* serogroup 1 (537/684 culture-confirmed HCA LD), the most common serogroups associated with culture-confirmed HCA LD were *L. pneumophila* serogroups 3 (33 cases), 6 (15 cases), and 5 (11 cases). Of the 107 community-acquired cases with culture confirmation of other *Legionella* species, 48 (44.9%) were caused by *L. longbeachae*. The European Surveillance System did not record any HCA cases of *L. longbeachae*.

**Table 3:** Causative pathogen of culture-confirmed, locally-acquired cases of Legionnaires’ disease, European Union–European Economic Area, 2008−2017

Species, serogroup	Community-acquired cases, no. (%)	Healthcare-associated cases, no. (%)
*Legionella pneumophila*		
1	3,600 (85.7)	537 (78.5)
2	22 (0.5)	3 (0.4)
3	126 (3.0)	33 (4.8)
4	15 (0.4)	7 (1.0)
5	15 (0.4)	11 (1.6)
6	52 (1.2)	15 (2.2)
7	11 (0.3)	0
8	12 (0.3)	2 (0.3)
9	3 (0.1)	0
10	16 (0.4)	8 (1.2)
11	0	0
12	1 (0.0)	2 (0.3)
13	1 (0.0)	
14	1 (0.0)	1 (0.1)
15	6 (0.1)	
Mixed	6 (0.1)	4 (0.6)
Non–serogroup 1*	15 (0.4)	8 (1.2)
Unknown	169 (4.0)	37 (5.4)
Subtotal *L. pneumophila* all serogroups	4,071 (96.9)	668 (97.7)
*L. anisa*	4 (0.1)	2 (0.3)
*L. bozemanii*	15 (0.4)	2 (0.3)
*L. cincinnatiensis*	1 (0.0)	0
*L. dumoffii*	4 (0.1)	2 (0.3)
*L. feeleii*	1 (0.0)	0
*L. longbeachae*	48 (1.1)	0
*L. macechernii*	2 (0.0)	0
*L. micdadei*	10 (0.2)	2 (0.3)
*L. sainthelensi*	1 (0.0)	0
*L. wadsworthii*	0	1 (0.1)
*Legionella* other species	21 (0.5)	4 (0.6)
Subtotal *L.* all other species	107 (2.5)	13 (1.9)
*Legionella* species unknown	22 (0.5)	3 (0.4)
Total	4,200 (100.0)	684 (100.0)

*Non–serogroup 1 refers to samples that do not belong to serogroup 1, but that do not have an identified serogroup

Of the 856 culture-confirmed cases caused by *L. pneumophila* serogroup 1 for which isolates were subtyped using mAbs, 679 (79.3%) tested positive for mAb 3/1 (Table 4). This proportion was higher for community-acquired than HCA LD (83.6% vs. 43.3%; p<0.01).

**Table 4:** Monoclonal subtype for *L. pneumophila* serogroup 1 isolates, European Union–European Economic Area, 2008−2017

Monoclonal subtype	Community-acquired cases, no. (%)	Healthcare-associated cases, no. (%)
Monoclonal antibody 3/1–positive*		
Allentown	4 (0.5)	1 (1.1)
Allentown/France	198 (25.8)	8 (8.9)
Benidorm	105 (13.7)	9 (10.0)
France	1 (0.1)	0
Knoxville	197 (25.7)	5 (5.6)
Philadelphia	135 (17.6)	16 (17.8)
Subtotal	640 (83.6)	39 (43.3)
Monoclonal antibody 3/1–negative		
Bellingham	38 (5.0)	11 (12.2)
Camperdown	4 (0.5)	0
Heysham	0	1 (1.1)
OLDA	26 (3.4)	15 (16.7)
Oxford	3 (0.4)	1 (1.1)
Oxford/OLDA	55 (7.2)	23 (25.6)
Subtotal	126 (16.4)	51 (56.7)
Total	766 (100.0)	90 (100.0)

*Monoclonal types are grouped by the presence (or lack) of the virulence-associated epitope recognized by the monoclonal antibody 3/1 (Dresden Panel) (*10*).

### Outcomes

Of the 32,379 case-patients with known outcomes, 3,448 (10.6%) died (Table 5). The proportion of patients who died was higher for those with HCA than community-acquired LD (28.8% vs. 8.6%; p<0.01). This proportion was similar for patients with hospital-acquired LD and LD linked to other healthcare facilities (29.2% vs. 28.1%; p = 0.52). After adjustment for age, sex, year, and reporting country, the death rate was higher for HCA than community-acquired LD (OR 3.02, 95% CI 2.75–3.32). The death rate was higher for hospital-acquired LD than for LD linked to other healthcare facilities (OR 3.50, 95% CI 3.14–3.91) (Table 5). After we restricted our analysis to the 4,121 culture-confirmed cases for which information was available about causative species and serogroups, the death rate for HCA LD remained higher than for community-acquired LD (OR 2.60, 95% CI 2.11–3.22). Patients infected by *L. pneumophila* nonserogroup 1 had a higher risk for death than those infected by *L. pneumophila* serogroup 1 (OR 2.17, 95% CI 1.61–2.92). Infection with other species was not associated with a higher death rate. Of the 690 culture-confirmed cases caused by *L. pneumophila* serogroup 1 for which information about monoclonal subtype was available, patients with HCA LD still had a higher death rate than those with community-acquired LD (OR 1.93, 95% CI 1.04–3.58); cases caused by mAb 3/1–negative strains were 4 times more likely to be fatal than those caused by mAb 3/1–positive strains (OR 4.20, 95% CI 2.32–7.61).

**Table 5:** Characteristics of locally acquired cases of Legionnaires' disease and adjusted predictors of death, European Union–European Economic Area, 2008−2017*

Characteristic	Survival, no. (%)	Death, no. (%)	Univariate logistic regression, OR (95% CI)	Multivariable logistic regression, OR (95% CI)†
Total	28,931 (100.0)	3,448 (100.0)		
Sex				
M	20,653 (71.6)	2,318 (67.4)	0.82 (0.76–0.89)	1.11 (1.02–1.21)
F	8,197 (28.4)	1,119 (32.6)	Referent	Referent
Unknown	81	11	Not included	Not included
Age at diagnosis, y				
<20	156 (0.5)	13 (0.4)	1.39 (0.78–2.47)	0.87 (0.44–1.72)
20–29	517 (1.8)	12 (0.3)	0.39 (0.22–0.69)	0.38 (0.21–0.68)
30–39	1,581 (5.5)	63 (1.8)	0.66 (0.51–0.87)	0.68 (0.52–0.89)
40–49	4,097 (14.2)	177 (5.1)	0.72 (0.60–0.86)	0.71 (0.59–0.86)
50–59	6,706 (23.2)	402 (11.7)	Referent	Referent
60–69	6,264 (21.7)	613 (17.8)	1.63 (1.43–1.86)	1.55 (1.35–1.76)
70–79	5,282 (18.3)	872 (25.3)	2.75 (2.43–3.12)	2.53 (2.23–2.87)
≥80	4,305 (14.9)	1,292 (37.5)	5.01 (4.45–5.64)	4.36 (3.85–4.93)
Unknown	23	4	Not included	Not included
Setting of infection				
Community	26,630 (92.0)	2,518 (73.0)	Referent	Referent
Hospital	1,534 (5.3)	631 (18.3)	4.35 (3.93–4.81)	3.50 (3.14–3.91)
Other healthcare facility	767 (2.7)	299 (8.7)	4.12 (3.59–4.74)	2.26 (1.94–2.63)

*OR, odds ratio. †Adjusted for year and reporting country.

### Clusters

Of the 31,973 LD patients with known cluster status, 2,039 (6.4%) were part of a cluster. This proportion was higher for HCA LD patients than community-acquired LD patients (10.6% vs. 6.0%; p<0.01).

## Discussion

In this surveillance sample from 29 EU–EEA countries, »10% of locally acquired LD cases were HCA. This analysis included >4,300 HCA LD cases reported during a 10-year period, providing a valuable overview of HCA LD epidemiology in Europe. A few countries accounted for most cases, a phenomenon that might limit the generalizability of the results (*2*,*20*). Although some countries might have more stringent preventive measures for hospitals, the characteristics of HCA LD patients themselves are unlikely to differ substantially across countries. In addition, we adjusted for the reporting countries in our statistical analyses. Most of the countries with a proportion of HCA LD >25% were also countries with low LD notification rates (<0.5 cases/100,000 population) during 2011–2015 (*2*). This finding suggests that these countries are better able to diagnose and report HCA than community-acquired LD cases. Some of these countries have reported challenges in ascertaining LD, including lack of clinical awareness, lack of testing, and lack of on-site diagnostic tests (*2*).

In Europe, HCA LD disproportionately affects older persons; 61.4% of case-patients are >70 years of age. However, HCA LD should not be overlooked in children. LD patients <20 years of age are twice as likely to have HCA LD than patients 50–59 years of age. Although the risk for HCA LD remains higher for men and boys than for women and girls (male:female ratio of 1.4:1), LD in female patients is 60% more likely to be HCA than it is in male patients. Some risk factors for community-acquired LD might be associated with sex. For example, activities that women might be less likely to engage in, such as home plumbing or working in transportation or construction, could be risk factors for LD (*21*,*22*).

The incidence of HCA LD varied less by season than it did for community-acquired LD, probably because healthcare facilities are less exposed to external environmental conditions. *Legionella* spp. often colonize hospital water systems (*23*). These water systems might offer year-round favorable conditions for *Legionella*, which multiplies at 25°C–42°C (*24*).

*Legionella* spp. causing HCA LD differ from those commonly observed in community-acquired LD. Although *L. pneumophila* caused most infections regardless of the setting, we observed a lower proportion of *L. pneumophila* serogroup 1 in HCA LD cases. This discrepancy may be of concern because UAT, the dominant laboratory method used to ascertain LD, has a poor sensitivity to non–*L. pneumophila* serogroup 1 strains (*25*). In our study, »45% of culture-confirmed cases were also ascertained by UAT. Because we could not determine whether the culture sequentially followed the UAT or whether the tests were performed independently, we might have overestimated the cases caused by *L. pneumophila* serogroup 1. Of the cases caused by *L. pneumophila* serogroup 1, mAb 3/1–negative strains were more common in HCA LD patients, whereas mAb 3/1–positive strains were more common in community-acquired LD patients. These results confirm earlier reports that mAb 3/1–negative strains were more frequent in hospital-acquired infections (*10*). The association of HCA LD with less virulent strains probably reflects patient demographic variables; immunocompromised patients might be more highly concentrated in healthcare facilities than in the general community. Although non–*L. pneumophila* species caused only a few cases, the proportion of cases caused by those species (except for *L. longbeachae*, which only causes community-acquired LD and is frequently associated with exposure to composts and potting soils [*26*]) was higher in patients with HCA than community-acquired LD. Patients with non–*L. pneumophila* infections might be more likely to be immunocompromised (*27*).

Nearly 30% of HCA LD patients in this analysis died. The risk for death was 2–3 times higher for HCA LD than for community-acquired LD. Some strains such as MAb 3/1–negative strains were also associated with a higher risk for death, probably because these strains of LD tend to infect more severely ill patients.

The HCA LD diagnosis might mask 2 different populations: younger but more severely ill patients who acquired infection in the hospital and older but less severely ill patients who acquired infection from other healthcare facilities. Hospital-acquired LD might be more likely to affect immunocompromised patients with underlying conditions. The large proportion of non–hospital-acquired LD in patients >70 years of age suggests that many might be residents of long-term care facilities. In these facilities, caretakers might have difficulty obtaining sputum samples or might not suspect LD. Furthermore, microbiology laboratory capacity might be limited (*28*), as suggested by the low proportion of culture-confirmed cases in these settings. 

There is no European standard for defining HCA LD. Epidemiologists in charge of national LD surveillance report LD cases with the probable setting of infection. These reports might misclassify LD patients who were infected in the community but admitted to the hospital during the incubation period (as reported in a patient with a possible incubation of >20 days [*29*]). Because the LD attack rate is very low, this situation is highly unlikely. In addition, epidemiologists classifying these cases might follow some definition (either national or not publicly available) for HCA LD, most likely on the basis of time between date of symptom onset and date of admission to hospital. Assuming equal rates of infection for both community-acquired and HCA LD, a study estimated that a cutoff of 6 days would identify HCA LD with a predictive value of >77% (*30*).

In conclusion, HCA LD cases are responsible for a large proportion of LD diagnoses in Europe and differ from community-acquired cases in many aspects, including demographic characteristics, causative pathogens, and outcome. Given the severity of the disease, officials must identify cases and control outbreaks as quickly as possible. An agreed-on case definition for HCA LD might streamline the surveillance process.
